# Probing the electronic transport on the reconstructed Au/Ge(001) surface

**DOI:** 10.3762/bjnano.5.159

**Published:** 2014-09-05

**Authors:** Franciszek Krok, Mark R Kaspers, Alexander M Bernhart, Marek Nikiel, Benedykt R Jany, Paulina Indyka, Mateusz Wojtaszek, Rolf Möller, Christian A Bobisch

**Affiliations:** 1 Smoluchowski Institute of Physics, Jagiellonian University, Reymonta 4, 30-059 Krakow, Poland; 2 Faculty of Physics, Center for Nanointegration Duisburg-Essen, University of Duisburg-Essen, 47048 Duisburg, Germany; 3 Faculty of Chemistry, Jagiellonian University, Ingardena 3, 30-060, 30-059 Krakow, Poland

**Keywords:** Au on Ge(001), electronic transport, multi probe STM, scanning tunnelling potentiometry

## Abstract

By using scanning tunnelling potentiometry we characterized the lateral variation of the electrochemical potential µ_ec_ on the gold-induced Ge(001)-c(8 × 2)-Au surface reconstruction while a lateral current flows through the sample. On the reconstruction and across domain boundaries we find that µ_ec_ shows a constant gradient as a function of the position between the contacts. In addition, nanoscale Au clusters on the surface do not show an electronic coupling to the gold-induced surface reconstruction. In combination with high resolution scanning electron microscopy and transmission electron microscopy, we conclude that an additional transport channel buried about 2 nm underneath the surface represents a major transport channel for electrons.

## Introduction

Structures consisting of single atoms represent the lower spatial limit for electronic circuits. On such a small scale, the electronic structure is dominated by quantum phenomena, i.e., the electronic conduction crucially relies on the electronic states. Recently, many studies focus on self-organized Au atom wires on the Ge(001) surface, which show Tomonaga–Luttinger liquid properties, i.e., represent a one-dimensional electronic system [1–3]. In contrast to other nanowire structures, e.g., in atoms on Si(111) [4] or Au on Si(557) [5], the Au/Ge(001) wires are rather robust against a Peierls distortion [6], so that the Au/Ge(001) surface offers the unique opportunity to study a Tomonaga–Luttinger liquid. In addition, such atomic scale wires may be used as atomic scale leads to contact, e.g., small atomic structures or molecules. The anisotropic transport properties of this surface structure have triggered controversial discussions within the scientific community [7–9]. However, to access the anisotropic transport properties, a significant electron current needs to be coupled to the atomic wires. At neighbouring terraces, the Au/Ge(001) wire structure is rotated by 90° and then a single layer step represents a domain boundary. Simultaneously, also the correlated electronic structure is rotated. Thus, the coupling between adjacent terraces can be probed by applying a lateral current through the reconstructed surface. Even though the metallic contacts to the Au/Ge(001) surface may be farther apart, a local sensitive probe can study the electronic properties in the vicinity of the domain boundaries. We know from previous experiments that the Au/Ge(001) surface exhibits a two dimensional conductance channel on a micrometre-scale averaging across several Au-reconstructed 1D domains [10].

**Figure 1:**
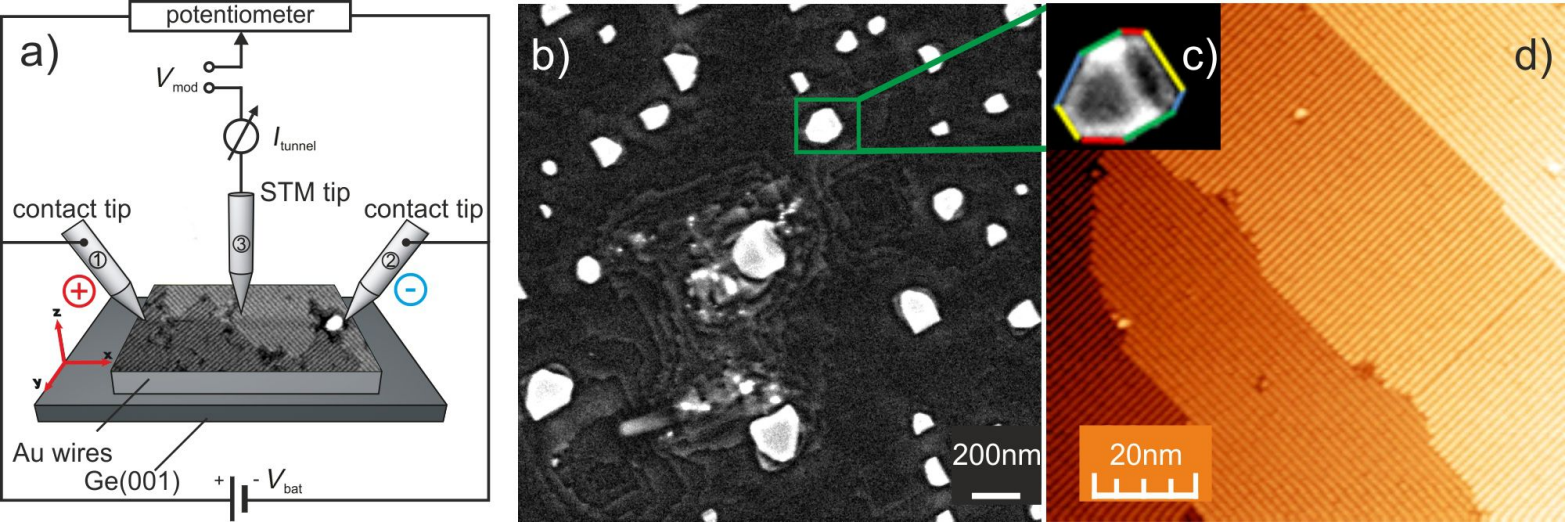
a) Scheme of the potentiometry setup using a multiprobe STM. Two STM tips (1 and 2) in contact with the sample surface are used to drive a lateral current. The third STM tip (3) simultaneously images the topography and the electrochemical potential of the surface. b) HRSEM image of the 6 ML Au/Ge(001) sample surface exhibiting the Au clusters and some terrace edges. Most clusters are of asymmetric octagonal shape as depicted by the colour contour in c). d) HRSTM image of an area between the Au clusters showing two differently oriented domains of the Au induced wires (*I*_T_ = 200 pA, *V_sample_* = −2 V).

Scanning tunnelling microscopy (STM) and various STM-based methods are excellent tools to study the topographic structure, the electronic structure, and electron transport phenomena of conducting surfaces at the limit of lateral resolution. By performing scanning tunnelling potentiometry (STP) [11] we tried to study the lateral variation of the electrochemical potential µ_ec_ (called potential in the following) at the boundary between two rotated Au wire-like domains while a lateral current was flowing through the Au/Ge(001) sample (see also the scheme in Figure 1a below). By using a multiprobe STM setup (Omicron Nanoprobe) individually controlled STM tips are used to establish well defined electric contacts to the reconstructed surface. We applied a voltage between two contacts leading to a current flow across the surface. Thus, if the main contribution of the total current is flowing through the Au reconstructed 1D domains, the impact of the predicted conductance anisotropy should be observed as a variation of the electrochemical potential in the vicinity of the domain boundaries.

## Experimental

The germanium substrate is cut from a wafer of a n-type Ge(001) crystal with a resistivity of about 30 Ω·cm. The cleaning procedure of the substrate consists of a few cycles of 600 eV Ar^+^ ion sputtering at a sample temperature of 1040 K (as measured by a pyrometer). After this procedure, the STM imaging proves that the Ge(001) surface exhibits atomically flat terraces with a lateral extension of 30–50 nm and a mixed (2 × 2)/c(4 × 2)-two domain reconstruction pattern as checked by low energy electron diffraction (LEED).

We deposited 6 monolayers (MLs) of Au on the reconstructed Ge(001) from a resistively heated crucible. The deposition rate of 0.2 ML/min is monitored by using a quartz crystal microbalance and the substrate temperature during the deposition of Au is kept at 150 K. After the deposition, no ordered structure is observed until the sample is annealed. After annealing to 770 K for about 10 min the Au-induced wire-like Ge(001)-c(8 × 2)-Au structure emerges [1,3]. The excess amount of Au aggregates into Au clusters. We intentionally deposited this excess amount of Au since the Au clusters may serve as metallic leads to contact the surface structure by STM tips [12–13]. In Figure 1b an overview of the sample surface is provided by a high resolution SEM image exhibiting several Au clusters together with the terrace edges. The Au clusters are of about 150 nm size and they are of an asymmetric octagonal shape at their base (see Figure 1b and Figure 1c). A high resolution STM image of the area between the Au clusters exhibits Au reconstructed terraces separated by single layer steps, exemplarily shown in Figure 1d. At most step edges the wire structure is rotated by 90° resulting from the reconstructed Ge(001) substrate. The domains exhibit some structural defects within the atomic wires. For the structural and electronic analysis of the samples two different experimental techniques were applied:

We use a multiprobe scanning tunnelling microscope (Nanoprobe by Omicron) to analyse the lateral variation of the potential caused by a current parallel to the surface. The mechanical stability and performance of the commercial STM setup was improved in order to provide atomic resolution, e.g., on the Si(111)-(7 × 7) surface or the Bi(111) surface [12]. During the scanning tunnelling potentiometry (STP) experiments, two tips contact the surface and drive a lateral current. A third STM tip simultaneously measures the topography and the potential of the surface between the contacts [13–15]. The STP experiments were carried out at a base pressure below 3 × 10^−10^ mbar for various sample temperatures between 130 K and 300 K. In order to establish smooth contacts to the surface, electrochemically etched Au tips were gently pressed against the Au/Ge(001) surface by sub-sequentially using the z-piezo drive of the STM unit for different course approach tip/sample separations. The contact formation is monitored for a bias voltage of 1 V between tip and substrate by the appearance of a contact current in the microampere regime. The lateral position of the STM tips is monitored by using a scanning electron microscope. The scheme of the STP measurement is depicted in Figure 1a: Two tips (1 and 2) contact the sample and apply a voltage *V*_bat_ leading to a transverse current *I*_trans_ through the surface while the third tip measures the STM topography and the potential, simultaneously. Therefore, a feedback loop adjusts the dc tunnelling voltage such that the dc tunnelling current becomes zero. Thus, for each lateral tip position the applied dc tunnelling voltage corresponds to the potential underneath the STM tip. This allows us to map the potential with atomic precision. To maintain a tunnelling distance between the STM tip and the sample surface, we additionally apply a small alternating tunnelling voltage (*V*_mod_) such that the tip/sample distance can be controlled by the corresponding ac component of the tunnelling current. Further experimental details can be found elsewhere [12,16]. The contact tips are placed such that the direction of the applied lateral current is mainly oriented orthogonal to the main direction of the germanium surface steps originating from the miscut of the Ge(001) sample. The contact area between the Au tips and the surface is relatively large, so we assume that both, the Au-induced wire-like Ge(001)-c(8 × 2)-Au structure and the Au islands are contacted by the tips simultaneously. All image acquisition was done by using the open source software GSxM [17] and data processing was done by using WxSM [18].

For the transmission electron microscope (TEM) measurements lamellas of the Au/Ge(001) of the very same sample were prepared with the use of an FEI Quanta 3D FEG scanning electron microscope equipped with a 30 keV Ga^+^ focused ion beam gun (FIB). In order to preserve the surface of the Au/Ge sample against the standard FIB operation during the lamella preparation, the sample surface at first was covered (capped) with a 20 nm layer of thermally evaporated carbon. Then, on top of the cap layer, a platinum layer was deposited using a gas injection system by the electron beam and the FIB beam was used to cut out the lamella. The high resolution (HR) TEM and high angle annular dark field (HAADF) scanning TEM images together with energy dispersive X-ray spectroscopy (EDX) analysis of the sample were obtained by the FEI Tecnai Osiris transmission electron microscope operated at 200kV electron beam.

## Results

**Figure 2:**
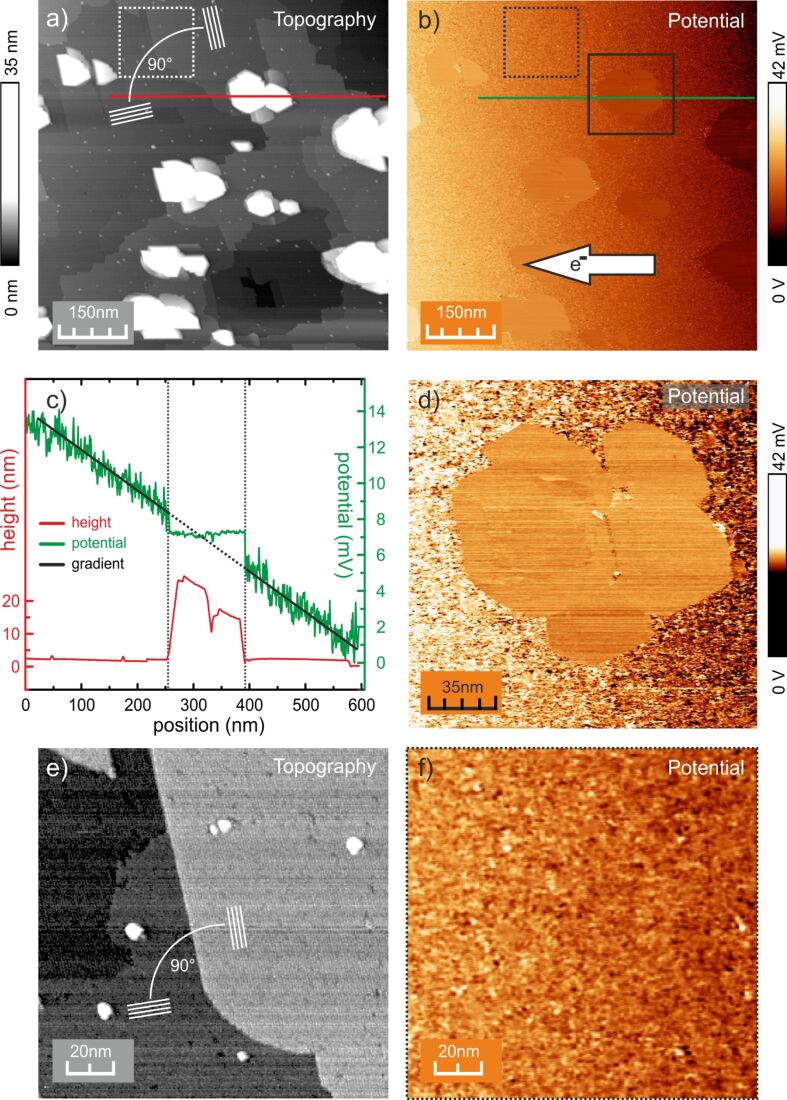
a) STM image of the Au/Ge(001) surface showing both different types of Au wire-like domains and several Au clusters. b) Corresponding image of the potential, exhibiting a constant gradient on the terraces (Au wire-like domains). The Au clusters appear with constant potential and rather sharp transitions at their borders. c) Line profiles of the topography (red) and potential (green) as marked in a) and b). As a guide to the eye a linear function was fitted to the overall gradient of the potential. d) Potential near a Au cluster. Sharp transitions of the potential at the perimeter of the cluster are easily recognized. e) and f) show the topography and the corresponding potential for a small area near a step edge, i.e., domain boundary. The directions of the atomic wires are marked by white lines in a) and e) (*V*_bat_ = 9 V, *I*_trans_ = 3 mA).

Figure 2a shows a large scale STM image of the Au/Ge(001) surface. Several surface steps and Au clusters are observed. The terraces exhibiting the Au wire-like structure are about 100 nm wide. In Figure 2a, for two of the terraces the corresponding directions of the wires are indicated by white lines. Due to the contact geometry the electrons are flowing in the direction indicated by the arrow in b). Hence, the current is oriented either approximately parallel or orthogonal to the wire-like structure, depending on the orientation of the domain. To ensure the best resolution for the potentiometry across (8 × 2) domain boundaries, i.e., step edges, the fast scan direction is chosen in parallel to the direction of the current.

A double-tip scan artefact is observed for the Au clusters. However, it can be well identified by the topography so that the corresponding potential was analysed accordingly. The potential shown in Figure 2b exhibits a gradient on the reconstructed terraces. In contrast, the potential on the Au clusters is rather constant at a value corresponding to a value of the surrounding terrace. The gradient of the potential of various STP images is determined to be about Δ*V* = 20 µV/nm for an estimated local average current density *j* of 11 A/m. The latter is estimated from the total transverse current *I*_trans_ and the contact geometry. In the middle of the connecting line between the contact tips, *j* can be written as [19]:


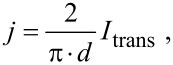


where *d* = 170 ± 20 µm is the distance between the contact tips and *I*_trans_ = 3 mA is the total transverse current. With these results and assuming an isotropic conductivity we can determine the conductivity σ = (*j*/Δ*V*) of the terraces' area at room temperature to be about 0.55 mS.

**Figure 3:**
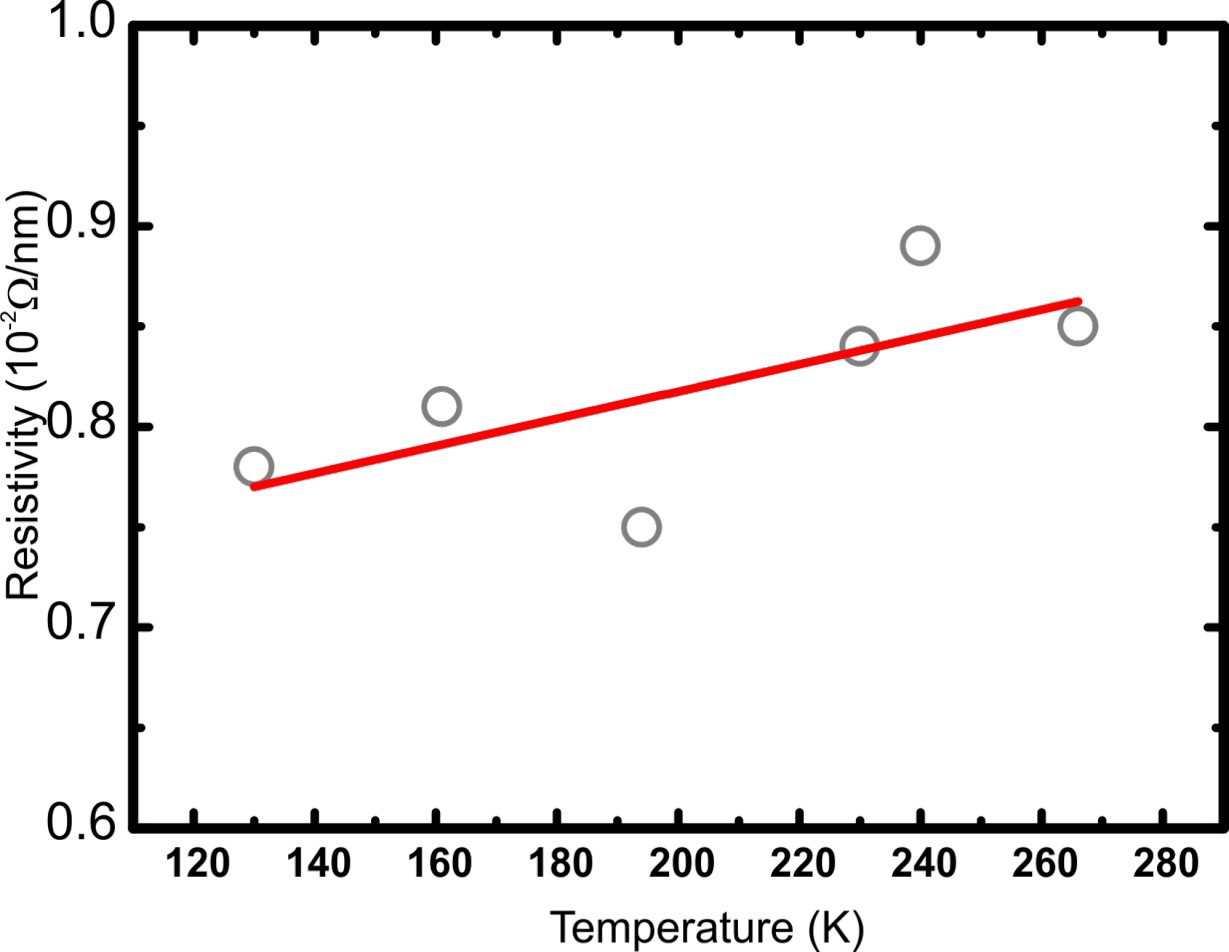
Resistivity, as evaluated from the gradient of the potential in the STP images, as a function of the temperature of the sample.

Figure 2c exemplarily shows line profiles of the potential and the corresponding topography as marked in Figure 2a and Figure 2b. On the reconstructed surface the potential exhibits strong fluctuations (±2.5 mV) but no direct correlation to steps in the topography. The potential on the Au clusters appears rather flat and smooth, only limited by the resolution of our STP setup (±5 µV) and is constant within the experimental error. At the edge of the Au clusters a discontinuity of the potential occurs. The resistivity, i.e., the corresponding gradient of the potential on the terraces scales about linearly as a function of the absolute sample temperature (Figure 3) indicating that the measured conductivity for the present Au/Ge(001) system is metallic. Although it may be assumed that the transverse current may heat the Ge sample, we do not see any indications for this in our data. As sample heating would result in a temperature difference between the tunnelling tip and the sample, a thermovoltage in the tunnelling gap would occur. This voltage would also be measured by our STP setup and would be independent of the polarity of the transverse current. Since we do not observe this effect, heating of the sample seems to be negligible.

**Figure 4:**
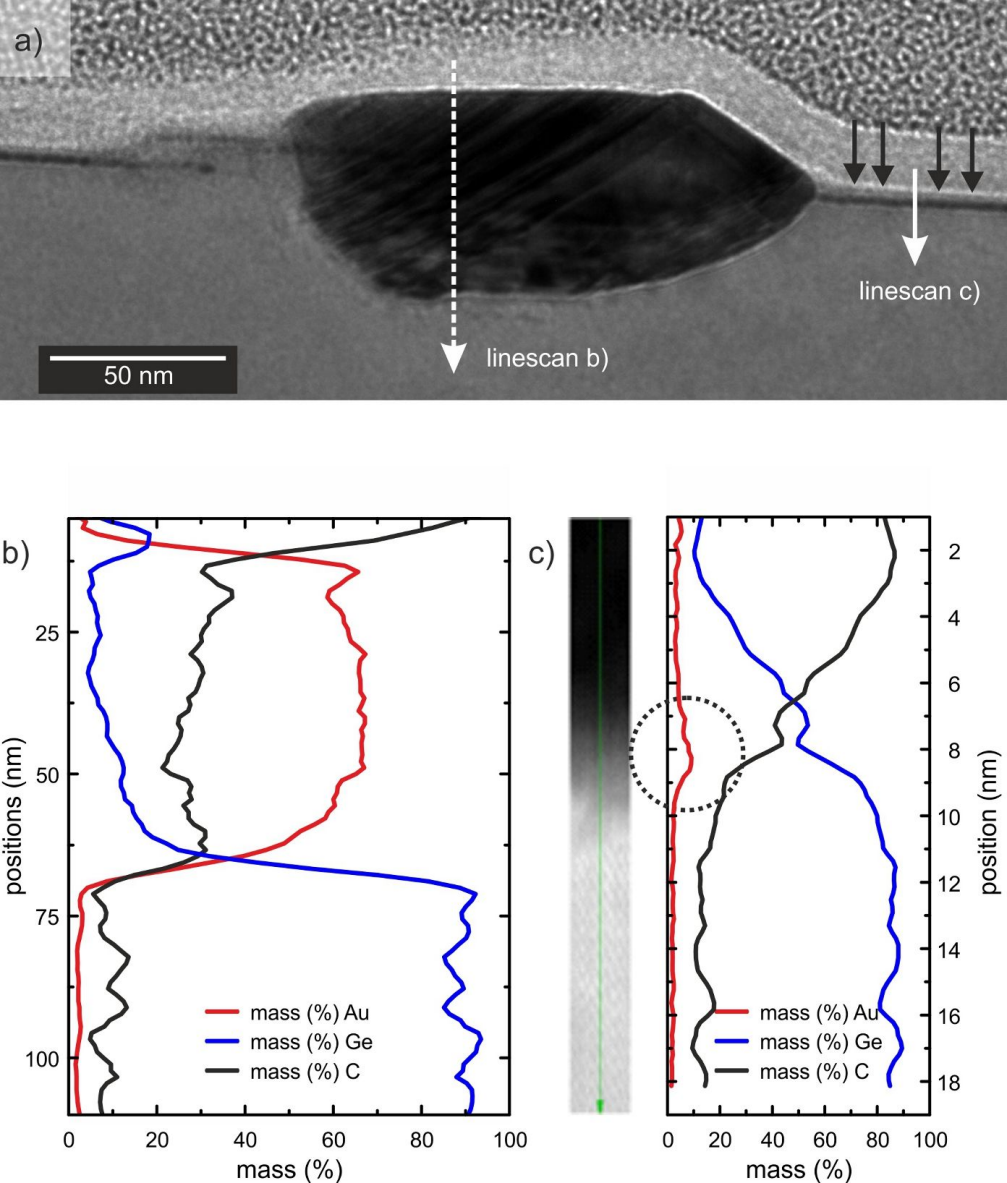
a) Exemplary TEM image of a lamella of Au grown on Ge(001) after the deposition of 6 ML of Au and annealing at 770 K. The apparent sample surface is exemplarily marked by black arrows on the right hand side of the image. b) EDX line scan analysis through the Au cluster. The Au cluster is reaching far into the Ge substrate. The non-zero Ge signal from the Au cluster is due to secondary fluorescence (excitations of “bulk” Ge caused by the X-ray emission from Au), which is a well-known effect (artefact) in the EDX spectroscopy. c) HAADF HRSTEM image through the Au/Ge lamella taken along the line on the right hand side in a). The results of the line scan EDX analysis are also shown. The Au concentration is found to reach its maximum of approx. 10% underneath the apparent sample surface (see circle). On the lower side of the image the atomic structure of Ge(001) bulk is visible.

In order to study the depth profile of the Au/Ge(001) samples, thin lamellas cut from the Au/Ge(001) sample were further analysed by means of high resolution TEM measurements. Figure 4 shows the corresponding TEM data. The contrast in the TEM image (Figure 4a) exhibits that the Au cluster (dark) is not only growing on top of the Ge surface, but also a large part of the cluster is digged into the Ge(001) substrate. The top surface of the cluster is not parallel to the Ge(001) substrate surface and is tilted by about 5° with respect to the substrate. This is common for all observed clusters. Furthermore, the measured angle between the side and top planes of the clusters (compare Figure 4a) is about 144.2 ± 1.6° which is very close to value of 144.8° corresponding to the angle between the [110] and [111] faces for a face-centred cubic crystal symmetry. These observations show that the excess amount of Au forms clusters of [110]-orientation, in agreement to previous STM studies of the same system by Wang et al. [20]. Also, HRTEM images with atomic resolution show that the Au clusters are crystalline.

Apart from that, in Figure 4a, a thin layer exhibiting the similar dark contrast as the gold cluster is also observed. This layer is extending from the cluster at both sides and is found about 2 nm below the apparent sample surface [21]. As a guide to the eye the apparent sample surface is marked by the black arrows in Figure 4a and determines the position where the grey contrast (Ge) changes into bright (capping carbon layer). The HAADF HRSTEM image (Figure 4c) through the Au/Ge lamella taken along the indicated line in a) and its corresponding EDX line profile show that this thin layer is enriched with Au. The occurrence of carbon throughout the whole observed lamella surface is due to the measurement process and only reflects the deposition and adsorption probability of C onto the different exposed materials along the surface of the lamella.

## Discussion

**Figure 5:**
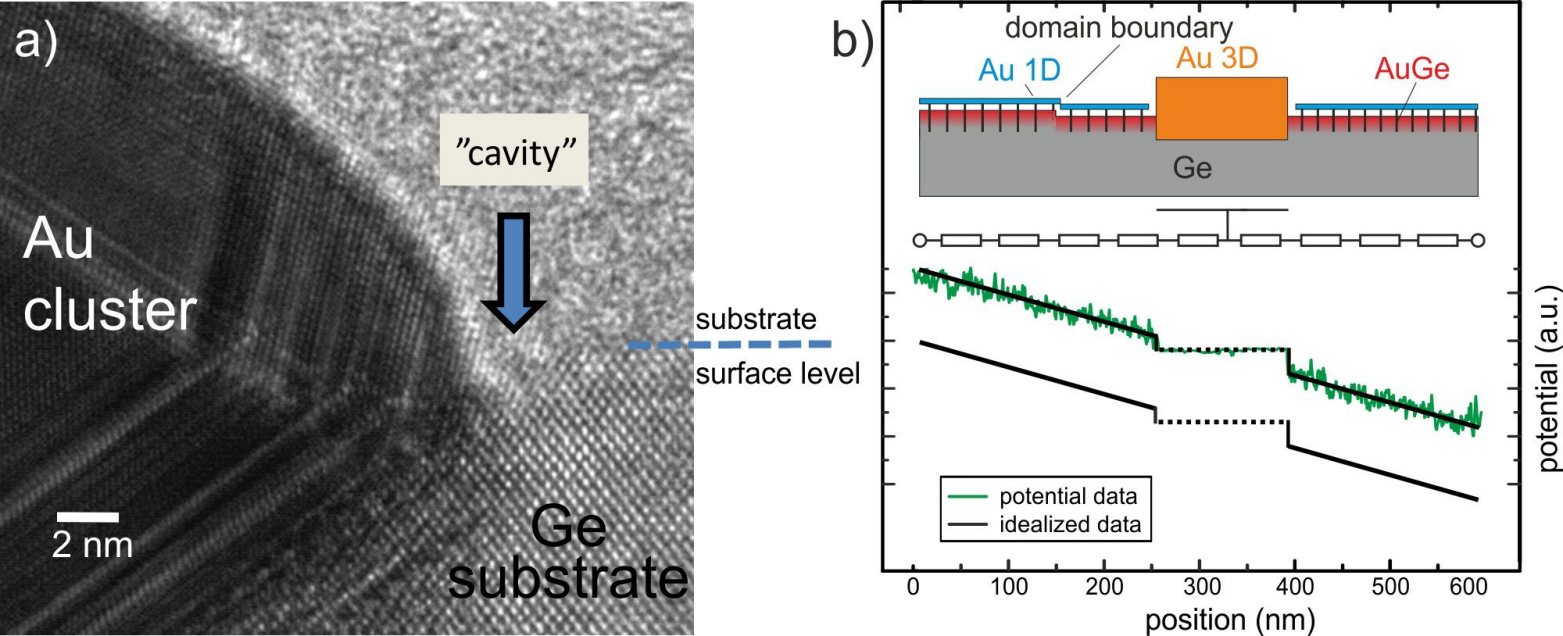
a) An atomically resolved HRTEM image of the interface between the Au cluster and the surrounding substrate surface. The substrate surface level is indicated with the dashed line. The arrow points to the discontinuity region (“cavity”) between the crystalline substrate surface region and the Au cluster. b) Scheme of the Au/Ge(001) sample structure. The Au cluster is electronically decoupled from the Au atomic wires but is coupled to a buried Au-enriched layer. The Au atomic wires are also coupled to this buried subsurface layer. The simplified wiring diagram is shown in the middle and a potential profile from original data and its simplified form is also shown.

We find an abrupt transition of the potential between Au clusters and the reconstructed Au/Ge surface which indicates that the clusters are not electrically coupled to the conducting channels of the Au-induced reconstructed Ge(001) surface. We have carefully checked that the discontinuity is not only caused by the double tip artefact. The abrupt transition from the linear slope on the terrace to the constant potential on the Au cluster appears for all observed Au clusters and at the perimeter of almost the whole cluster. A careful inspection reveals that there exists one direction, along which the potential on the cluster matches the potential on the surrounding terrace. Since the potential on the Au clusters is constant for the whole area of the cluster the potential on the Au clusters is not caused by a tip artefact. It may be possible that the variation of the potential for the flat surface and the Au clusters occurs on a scale which is much smaller than the topographic and potential resolution of the experimental setup. Also in this case an abrupt variation of potential would be observed. However, we explain our findings by a two dimensional conducting layer underneath the surface which is electronically coupled to both, the Au atomic wires and the Au clusters while the Au clusters and the Au atomic wires at the surface are not coupled to each other. Our HRTEM data supports this assumption. In Figure 5, an atomically resolved HRTEM image of the interface between the Au cluster and the substrate surface is shown. In image a), on the right side the substrate surface level is indicated by a dashed line. It is clearly seen that the substrate surface region does not propagate with crystalline order to the Au cluster. A discontinuity region (about 2 nm wide), called in the image “cavity”, may either be a substrate depletion filled with carbon or disordered germanium. In both cases, this results in a weak electrical connection between the cluster and the reconstructed Au/Ge terraces.

The occurrence of such a Au-enriched layer is not unexpected since Au is known to segregate into Ge bulk [22] especially at elevated temperatures. Therefore, we conclude that the subsurface layer emerges upon preparation of the Au/Ge(001) sample. By applying a voltage between the contacts to the surface, the current can also flow through the buried Au-enriched layer. Since the step edges, i.e., domain boundaries are expected to be scattering centres for the current, some contrast in the potential similar to surface transport in Si(111)-√3 × √3:Ag [13] and thin Bi(111) films on silicon [12,16] would be expected. However, the maps of the potential show no fine-structure related to the step edges or other surface defects so we conclude that the main current is not carried by the surface, i.e., the Au atomic wires, but by the subsurface layer. Thus, the corresponding conductivity of the buried layer is higher than the conductivity along the sample surface including the Au atomic wire structure, the Au wire domain boundaries and the interface between the Au clusters and the Au wire domains. A simple model for the Au/Ge structure is shown in Figure 5b. In addition, a line profile of the potential across a Au cluster and an idealized profile for the depicted simple wiring scheme is shown.

**Figure 6:**
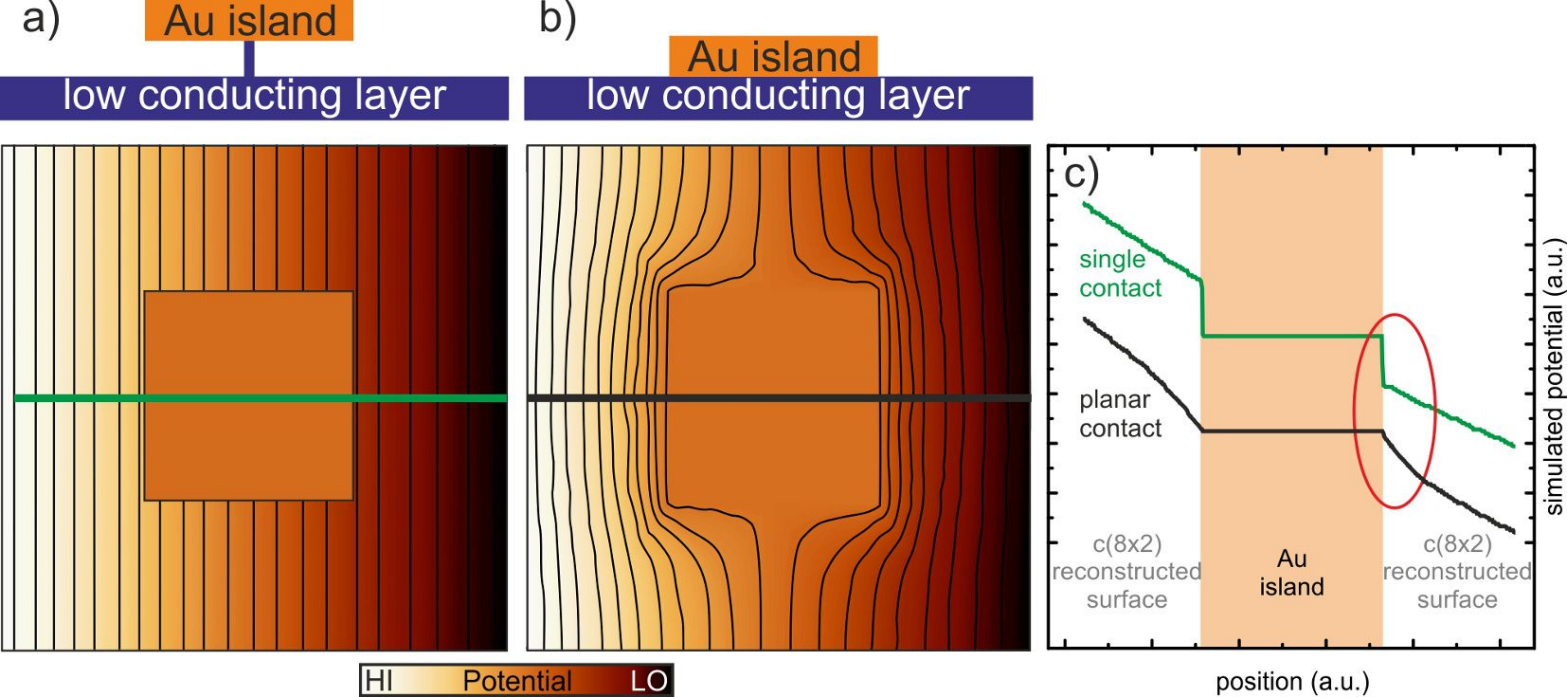
a) Finite elements simulation for the potential if one single path connects a highly conductive cluster to a low conducting substrate while the borders of the cluster are not connected to the substrate (single point contact). b) Simulated potential if the highly conductive cluster is placed directly on top of a low conducting substrate (planar contact). The colour palette represents the potential variation. As a guide to the eye equipotential lines are superimposed. A side view of the corresponding contact geometry is shown above each simulation. c) Line profiles for both contact geometries in a) and b); the upper graph/profile exhibits a sharp transition, while the lower graph exhibits a bending of the potential in the proximity of the cluster.

To test our hypothesis we performed a simple finite elements simulation for a comparable conductive structure by using FEMLAB [23]. Figure 6 shows two simulated images of the potential which show that the sharp transition at the Au clusters can be simulated if a highly conductive cluster is placed on a lower conductive material. If no metallic contacts are present at the perimeter of the cluster and only a single point contact underneath the cluster (Figure 6a) is active, a sharp transition similar to the findings in our potentiometry data is found. As a guide to the eye, equipotential lines are plotted which show the impact of the cluster on the potential in its vicinity.

Our potentiometry data correspond quite well to Figure 6a, which corroborates our assumption for the sample structure as depicted in Figure 5b. If instead the cluster is placed onto the low conductive material with a planar contact a smoother transition occurs and the potential bends towards the cluster edges (see Figure 6b). Line profiles for both cases are shown in Figure 6c.

## Conclusion

In conclusion, we find that the electronic transport properties of the system Au/Ge(001) are not only given by the atomic wire-like surface structure exhibiting a Tomonaga–Luttinger behavior, but also by a 2D conductive layer underneath the surface. Upon contacting the Au/Ge(001) sample surface, we contact the surface structure and the subsurface layer which both carry the resulting electric current. Since no lateral variations of the potential are observed in the vicinity of domain boundaries at the Au induced wire-like Ge(001)-c(8 × 2)-Au structure, we conclude that the subsurface layer appears to be the major transport channel for this contact geometry. Rather sharp transitions of the potential at embedded Au islands suggest a decoupling of the Au islands from the surface layer. From in depth-profile analysis we can conclude that the Au islands contact the Au enriched subsurface layer which carries the lateral current. Therefore, the peculiar electronic structure of the Au/Ge(001) surface is not accessible even if micrometre-sized point contacts to the Au/Ge surface are used. These findings are of major importance if the Au/Ge(001) atomic wire structure shall be contacted by metallic leads to access its one-dimensional transport properties. The appropriate choice of electric leads appears to be a crucial parameter for passing electric currents through the one-dimensional electronic structure of Ge/Au. This may have wider impact, since segregation needs to be considered for other atomic wire-like surface structures as well. Whenever surface structures are engineered by adsorbing material, in depth profile analysis may unravel buried electronic channels which can prevent to access to the electronic system of the surface.

## References

[1] Haldane F D M (1981). J Phys C.

[2] Schäfer J, Blumenstein C, Meyer S, Wisniewski M, Claessen R (2008). Phys Rev Lett.

[3] Blumenstein C, Schäfer J, Mietke S, Meyer S, Dollinger A, Lochner M, Cui X Y, Patthey L, Matzdorf R, Claessen R (2011). Nat Phys.

[4] Yeom H W, Takeda S, Rotenberg E, Matsuda I, Horikoshi K, Schaefer J, Lee C M, Kevan S D, Ohta T, Nagao T (1999). Phys Rev Lett.

[5] Segovia P, Purdie D, Hengsberger M, Baer Y (1999). Nature.

[6] Blumenstein C, Schäfer J, Morresi M, Mietke S, Matzdorf R, Claessen R (2011). Phys Rev Lett.

[7] Heimbuch R, Kuzmin M, Zandvliet H J W (2012). Nat Phys.

[8] Nakatsuji K, Komori F (2012). Nat Phys.

[9] Blumenstein C, Schäfer J, Mietke S, Meyer S, Dollinger A, Lochner M, Cui X Y, Patthey L, Matzdorf R, Claessen R (2012). Nat Phys.

[10] Wojtaszek M, Kolmer M, Godlewski S, Budzioch J, Such B, Krok F, Szymonski M, Joachim C (2012). Multi-Probe Characterization of 1D and 2D Nanostructures Assembled on Ge(001) Surface by Gold Atom Deposition and Annealing. Atomic Scale Interconnection Machines.

[11] Muralt P, Pohl D W (1986). Appl Phys Lett.

[12] Bannani A, Bobisch C A, Möller R (2008). Rev Sci Instrum.

[13] Homoth J, Wenderoth M, Druga T, Winking L, Ulbrich R G, Bobisch C A, Weyers B, Bannani A, Zubkov E, Bernhart A M (2009). Nano Lett.

[14] Druga T, Wenderoth M, Homoth J, Schneider M A, Ulbrich R G (2010). Rev Sci Instrum.

[15] Muralt P, Meier H, Pohl D W, Salemink H W M (1987). Appl Phys Lett.

[16] Bobisch C A, Möller R (2012). Chimia.

[17] Zahl P, Bierkandt M, Schröder S, Klust A (2003). Rev Sci Instrum.

[18] Horca I, Fernández R, Gómez-Rodríguez J M, Colchero J, Gómez-Herrero J, Baro A M (2007). Rev Sci Instrum.

[19] Ji S-H, Hannon J B, Tromp R M, Perebeinos V, Tersoff J, Ross F M (2012). Nat Mater.

[20] Wang J, Li M, Altman E I (2005). Surf Sci.

[R21] 21To ensure a correct estimation of the buried layer depth we did also EFTEM (energy filtered TEM) and EELS measurements; all data support that the Au layer is buried around 2 nm below the Ge sample surface.

[22] Dornath-Mohr M A, Cole M W, Lee H S, Wrenn C S, Eckart D W, Fox D C, Yerke L, Chang W H, Lareau R T, Jones K A (1990). MRS Online Proc Libr.

[R23] 23FEMLAB. http://www.math.chalmers.se/Math/Research/Femlab/ (accessed May 15, 2014).

